# Novel interactions between ERα-36 and STAT3 mediate breast cancer cell migration

**DOI:** 10.1186/s12964-019-0409-4

**Published:** 2019-08-13

**Authors:** Yuan Xiang, Jia Peng Li, Wei Guo, Dan-Qun Wang, Ao Yao, Hui-Min Zhang, Feng Huang, Han-Han Li, Zhou-Tong Dai, Zi-Jiang Zhang, Hui Li, Yao Tan, Kun Chen, Le-Yuan Bao, Xing-Hua Liao

**Affiliations:** 1 Institute of Biology and Medicine, Wuhan University of Science and Technology, Wuhan, Hubei 430081 China; 2 Shenzhen Ritzcon Biological Technology Co., LTD, Shenzhen, Guangdong 518000 China; 3 Yiling Hospital, Yichang, Hubei 443000 China; 4 The Affiliated Tumor Hospital of Xinjiang Medical University, Uygur Autonomous Region, Urumqi, Xinjiang 830011 China; 5 College of Pharmaceutical, Liaocheng University, Liaocheng, Shandong 252000 China

**Keywords:** ERα-36, STAT3, Breast cancer, Migration

## Abstract

**Background:**

Breast cancer is the leading cause of cancer death in women worldwide which is closely related to metastasis. But the exact molecular mechanism of ERα-36 and STAT3 on metastasis is still not fully understood.

**Methods:**

MCF-7 and MDA-MB-231 human breast cancer cell lines and MCF-10A were overexpressioned or knockdown ERα-36 and STAT3 and tested for migration, invasion and proliferation assays. Direct interaction of STAT3 and ERα-36 were analyzed by coimmunoprecipitation assays. The effect of STAT3 and ERα-36 on MMP2/9 expression was analyzed by qPCR and western blotting. STAT3 phospholyation and acetylation by ERα-36 and p300 were observed and quantified by coimmunoprecipitation assays and western blotting.

**Results:**

Cross-talk between ERα-36 and STAT3 was demonstrated to mediate through a direct physical association between the two proteins. Furthermore, the interaction between ERα-36 and STAT3 was demonstrated to give rise to functional changes in their signaling events. Both MMP2 and MMP9 expression require the binding of the newly identified protein complex, ERα-36-STAT3, to its promoter, the second phase, which is more robust, depends on ERα-mediated recruitment of p300 onto the complex and the subsequent acetylation of STAT3. In addition, STAT3 is tyrosine-phosphorylated in a biphasic manner, and the late phase requires ERα-36-mediated p300-dependent acetylation. Furthermore, interference with acetylation of STAT3 by overexpression of acetylation null STAT3 mutant led to the loss of MMP2 and MMP9 expression. ChIP analysis and reporter gene assays revealed that ERα-36-STAT3 complex binding to the MMP2 and MMP9 promoter led to an enhanceosome formation and facilitated MMP2 and MMP9 expression.

**Conclusions:**

Our studies demonstrate for the first time that the function of MMP2 and MMP9 in breast cancer cell migration, which is mediated by interactions between ERα-36 and STAT3.

## Background

Estrogen exerts most of its biological effects through the estrogen-specific transcription factor, the estrogen receptor (ER) [1]. The estrogen activated ERs form dimers and subsequently associate with specific consensus DNA sequences called estrogen response elements (ERE) located in the promoter regions of target genes to regulate gene transcription. ER is a member of the nuclear receptor super family and shares the common protein structure of this family [2, 3]. The protein can be divided into several functional domains, denoted as A to F from the N terminus to the C terminus. The most conserved DNA-binding domain (DBD) C is comprised of two distinct zinc fingers and is responsible for DNA-binding and protein dimerization. The C-terminal E domain or ligand binding domain (LBD) plays important roles in mediating ligand binding, receptor dimerization, nuclear translocation, and ligand-dependent transactivation (AF-2) of target gene expression. The N-terminal A/B domain is highly variable in both sequence and size. It usually contains a transactivation function (AF-1), which activates target genes by directly interacting with components of the core transcription machinery or with coactivators that mediate signaling to the downstream proteins. The hinge domain D contributes flexibility to the DBD versus LBD and has also, in some cases, been shown to influence the DNA-binding properties of individual receptors, thus serving as an anchor for certain co-repressor proteins. Finally, the C-terminal F domain has been shown to contribute to the transactivation capacity of the receptor [4]. But its other functions, if any, are to a large extent unknown.

ERα-36 is generated from a promoter located in the first intron of the ERα-66 gene and differs from the ERα-66 by lacking both transcriptional activation domains (AF-1 and AF-2) but retaining the DNA-binding and dimerization domains, and partial ligand-binding domains [5, 6]. ERα-36 was reported to be a novel membrane associated ER that mediates rapid estrogen and anti-estrogen signaling in both ER-positive and -negative breast cancer cells predominantly expressed outside the cell nucleus and at the plasma membrane of both ER-positive and -negative breast cancer cells, mediates rapid estrogen and anti-estrogen signaling such as activation of the MAPK/ERK and PI3K/AKT signaling pathways and stimulation of cell proliferation [6, 7].

STATs are a family of transcription factors that consist of seven members. They contain a dimerization domain, a coiled-coil domain, a DNA-binding domain, a src homology 2 domain, and a conserved single tyrosine residue whose phosphorylation is required for its activation in the N-terminal region, whereas a transcriptional activation domain is present in the C-terminal region [8]. Interestingly, the STATs are the only characterized transcription factors thus far that are activated by tyrosine phosphorylation, and this post-translational modification inessential for their Src homology 2-mediated homo- or heterodimer formation [9, 10].

Our previous and other studies have shown that STAT3 promoted breast cancer cell migration, and MRTF-A and STAT3 synergistically increased MDA-MB-231 cell migration by promoting the expression of migration markers Myl-9, Cyr-61, urokinase-type plasminogen activator (uPA) and osteopontin (OPN) and inhibiting the expression of breast cancer metastasis suppressor 1 (BRMS1, [11, 12]). And MiR-93-5p inhibited the EMT of breast cancer cells via targeting MKL-1 and STAT3 [13]. Thus, it is interesting to note that although both ERα-36 and STAT3 belong to two different groups of transcription factors and are activated by opposite post-translational modifications; both target the two genes, MMP2 and MMP9, in mediating cell proliferation and migration [14–16].

It has been reported that MMP2 and MMP9 were closely related to cells migration in various cancers including bladder cancer, osteosarcoma, hepatocellular carcinoma, thyroid cancer, gastric adenocarcinoma [17–20], which are mediated by many signal pathways including MAPK/P38 signaling [21, 22], PI3K/AKT signaling [23, 24], Wnt/Frizzled signaling [25] accompanied by epithelial to mesenchymal transition (EMT), drug resistance [26, 27] and tumor progression. Because MMP2 and MMP9 play a critical role in cell cycle progression, it is not surprising that cells employ multiple mechanisms in its regulation in response to a plethora of stimuli [16, 28]. However, what is not known is whether there is any cross-talk among the multiple transcription factors that are activated by various signaling mechanisms in the induction of MMP2 and MMP9 expression in mediating cell cycle progression and cell migration in response to an agonist. Toward this end, we report here for the first time that ERα-36 and STAT3, members of two different families of transcription factors, exist as a heterodimer in quiescent cells and in response to thrombin bind to the MMP2 and MMP9 promoter in a cooperative and biphasic manner. In addition, ERα-36 facilitates STAT3 acetylation via recruiting p300 association. In response to IL-6, STAT3 is tyrosine-phosphorylated in a biphasic manner in which the late phase phosphorylation requires ERα-36-dependent p300-mediated acetylation. Furthermore, the cooperative interaction between these two transcription factors lead to a biphasic expression of MMP2 and MMP9, with the first phase influencing cell migration.

## Materials and methods

### Reagents

Recombinant human interleukin 6(IL-6), AG490, estradiol (E2) and tamoxifen (TAM) protein were obtained from R&D Systems and Sigma. Antibodies against MMP2, MMP9, p300, ERα-36, and STAT3 were purchased from Cell Signaling and Abcam; Myc, Flag and HA antibody were purchased from proteintech.

### Cell lines

MCF-7 and MDA-MB-231 human breast cancer cell lines and MCF-10A were grown in Dulbecco’s modified Eagle’s medium (DMEM)(GIBCO) supplemented with 10% fetal bovine serum at 37 °C in a 5% CO_2_ incubator.

### Plasmids

The pCDNA3.1-ERα-36-Myc contained a cDNA encoding amino acids 1–311 of human ERα-36, the pCDNA3.1-STAT3-Flag contained a cDNA amino acids 1–769 of human STAT3. The vector pCDNA3.1 was purchased from Promega. The promoter regions of MMP2 (− 904/+ 50) and MMP9 (− 4136/+ 18) were amplified by PCR and then were cloned into pGL3 luciferase reporter vector, respectively. The primers used to create MMP2-luc and MMP9-luc was as follow: MMP2: 5′- GTCAGCTAGCAGAGACGGTTGTCACAGGGA − 3′(sense) and 5′- AGACCTCGAGTGCTACTCCTGGCCTCTACG − 3′(antisense); MMP9: 5′- GTCAGCTAGCGCTTCCTTACTGACGGTGCT − 3′(sense), and 5′- AGACCTCGAGGGTGAGGGCAGAGGTGTC − 3′(antisense); the vector pGL3(Pomega) was used as a control. Additional luciferase reporter constructs of MMP2 and MMP9 promoter containing mutations to putative GAS were generated using the QuickChange site-directed mutagenesis kit (Stratagene, La Jolla, CA). The MMP2-WT-luc GAS was mutated from -TTCGTGGAA - to -TAGACGTAA -(MMP2-M-GAS-luc) and these nucleotide mutations abolished STAT3-binding sites. The primers used were as follows: MMP2-M-GAS-luc: forward 5′: GGGGAATAGACGTAACTGAGGGCTCCTCCCCTTTTTAGACCATATA 3′; reverse 5′: CCTCAGTTACGTCTATTCCCCACTCACTCTTGAATCCTTTCCTGCG 3′. The MMP9-WT-luc GAS was mutated from - TTCCCCTGAA - to - TAGATCGTAA - (MMP9-M-GAS-luc) and this nucleotide mutations abolished STAT3-binding sites. The primers used were as follows: MMP9-M-GAS-luc: forward 5′: TTGCCATAGATCGTAAGCTAGGGATGGCAGGTGTGTTTGTGGGTTG 3′; reverse 5′: CCTAGCTTACGATCTATGGCAATTCCCACACTGACTCTTAGAGTCC 3′.

### Wound-healing assays

For wound healing assays, cells were seeded on 6-well dishes. Twenty-four hours after transfection, wounding was induced by scratching the monolayer with a micropipette tip and the dish was placed at 37 °C in a 5% CO_2_ incubator chamber. Pictures were acquired after 0 h and 24 h (in the case of MDA-MB-231) or 36 h (in the case of MCF-7) using a microscope.

### Transwell migration assays

Cell migration was tested in 8-μm-pore polycarbonate membrane Transwell chambers (Corning) essentially as described previously [12, 13, 29, 30]. Cells were resuspended in DMEM/F12 without FBS (fetal bovine serum), and 75,000 cells were added to the top chamber of the Transwell chambers. DMEM/F12 containing 10%FBS was added to the bottom chamber, and cells were allowed to migrate for 24 h. Non-migrated cells were scraped off the top of the membrane. Migrated cells were fixed in 4% formaldehyde and stained in Giemsa. Cells were counted under a microscope in five different fields in duplicate wells, in at least three independent experiments.

### Reverse-transcription polymerase chain reaction (RT-PCR) and quantitative real-time RT-PCR (qRT-PCR)

RT-PCR and qRT-PCR analysis were carried out as described previously [29, 30]. Briefly, total cellular RNA was extracted from cultured cells with Trizol reagent (Invitrogen) and 2 μg of total RNA was reverse-transcribed using M-MLV reverse transcriptase (Promega) according to the manufacturer’s instructions. The thermal cycle profile was as follows: denaturation for 30 s at 95 °C, annealing for 45 s at 52–58 °C depending on the primers used, and extension for 45 s at 72 °C. Each PCR reaction was carried out for 28–32 cycles. PCR products were visualized on 2% agarose gels stained with ethidium bromide (EB) under UV trans-illumination. qRT-PCR was performed in an Applied Biosystems StepOne™ Real-Time PCR System. Fast SYBR®Green Master Mix was obtained from Applied Biosystems. Data were shown as relative expression level after being normalized by Glyceral-dehyde-3-phosphate dehydrogenase (GAPDH). The PCR primer sequences are as follows, GAPDH: F-5’ATTCAACGGCACAGTCAAGG3’, R-5’GCAGAAGGGGCGGAGATGA3’; MMP2:F-5′ CCCACTGCGGTTTTCTCGAAT 3′, R-5′ CAAAGGGGTATCCATCGCCAT 3′;MMP9:F-5′ AGACCTGGGCAGATTCCAAAC 3′, R-5′ CGGCAAGTCTTCCGAGTAGT 3′;STAT3:F-5’CAGCGAGAGCAGCAAAGAAG3’, R-5’GGAATGTCGGGGTAGAGGTAG3’.

### Western blotting

Western Blotting was performed as described previously [11, 30]. The total proteins of treated and tranfected cells were immunoblotted with rabbit p300, ERα-36 (Abcam), mouse phosphorylated STAT3 (Abcam), rabbit STAT3 (Abcam), rabbit MMP2(Abcam), mouse MMP9 (Abcam) and mouse p53, β-tubulin, and GAPDH (Santa Cruz) antibodies overnight at 4 °C, and then incubated with IR Dye™-800 conjugated anti-rabbit secondary antibodies and IR Dye™-680 conjugated anti-mouse secondary antibodies (Li-COR) for 30 min at room temperature (RT). The specific proteins were visualized by Odyssey™ Infrared Imaging System (Gene Company). GAPDH expression was used as an internal control to show equal loading of the protein samples.

### Immunofluoresence staining

Immunocytochemistry assays were performed as described previously [11, 31]. The cells after treatment were fixed in 4% paraformaldehyde for 15 min, and then blocked with normal goat serum for 20 min. Then, rabbit MMP2(1:200 dilution, Abcam), and mouse MMP9(1:200 dilution, Abcam) antibodies were added and incubated in a humid chamber over night. After washing with PBS twice, cells were incubated with appropriate secondary antibodies (fluorescein isothiocyanate (FITC)-goat anti-rabbit or anti-mouse IgG, SantaCruz) for 30 min at 37 °C. After washing with PBS, the samples were observed under laser scanning confocal microscope (Olympus). 4′,6-diamidino-2-phenylindole (DAPI) stain (blue) highlights the total nuclei.

### Luciferase reporter assays

Luciferase assays were performed as described previously [12, 31]. 24 h after transfection, luciferase activity was measured by on a Synergy™ 4 (Bioteck). Transfection efficiencies were normalized by total protein concentrations of each luciferase assay preparations. All experiments were performed at least three times with different preparations of plasmids and primary cells, producing qualitatively similar results. Data in each experiment are presented as the mean ± standard deviation of triplicates from a representative experiment.

### Chromatin immunoprecipitation (ChIP) assay

ChIP analysis was performed using a commercially available kit (Enzymatic Chromatin IP (Magnetic Beads), Cell Signaling Technology). MDA-MB-231 cells were transfected with STAT3 and ERα-36/STAT3 for 24 h. Proteins bound to DNA were cross-linked using formaldehyde at a final concentration of 1% for 20 min at room temperature. Protein-DNA complexes were immunoprecipitated using primary antibodies for ERα-36 (Abcam). STAT3 and MMP2 promoter complexes and STAT3 and MMP9 promoter complexes were measured by PCR. The primers used for the amplification of the human MMP2 promoter was: MMP2-GAS-box: forward-5′ AGACGGTTGTCACAGGGAG 3′, and reverse-5′ ATGGCAATGTGGGGAGGT 3′. And the primers used for the amplification of the human MMP9 promoter was: MMP9-GAS-box: forward-5′ TGACTTGGTGTTAATAGGGACT 3′, and reverse-5′ GGCAGGATGTGGAGGAGA 3′. The samples were electrophoresed using a 2% agarose gel, and visualized by ethidium bromide staining.

### Coimmunoprecipitation

Plasmid expressing Myc-ERα-36 and Flag-STAT3 were cotransfected into MDA-MB-231 cells. Lysates were collected 48 h posttransfection, and Myc antibody (Protein tech) was used to precipitate Myc-ERα-36 or Flag antibody (Protein tech) was used to precipitate Flag-STAT3, respectively. The resulting mixture was washed, subjected to sodium dodecyl sulfate-polyacrylamide gel electrophoresis (SDS-PAGE), transferred to a NC membrane (Pall), first revealed by Myc antibody (Protein tech) to visualize Myc epitoped ERα-36 or Flag antibody (Protein tech) to detect Flag epitoped STAT3, and then reprobed with Flag antibody (Protein tech) to detect Flag epitoped STAT3 or reprobed with Myc antibody (Protein tech) to detect Myc epitoped ERα-36, respectively.

### Statistical analysis

Statistical analyses were conducted using Statistical Program for Social Science (SPSS) software 17.0 (SPSS, USA). Percentages and ratios were arcsine transformed prior to data analysis. A 2-sided *P* value of 0.05 was considered statistically significant. Comparisons between 2 groups were analyzed by Student’s t-test. Data are presented as mean ± SD unless otherwise stated.

## Results

### IL-6 induces stat3 phosphorylation and mediates breast cancer cell migration through regulating MMP2/9 expression

**Fig. 1:**
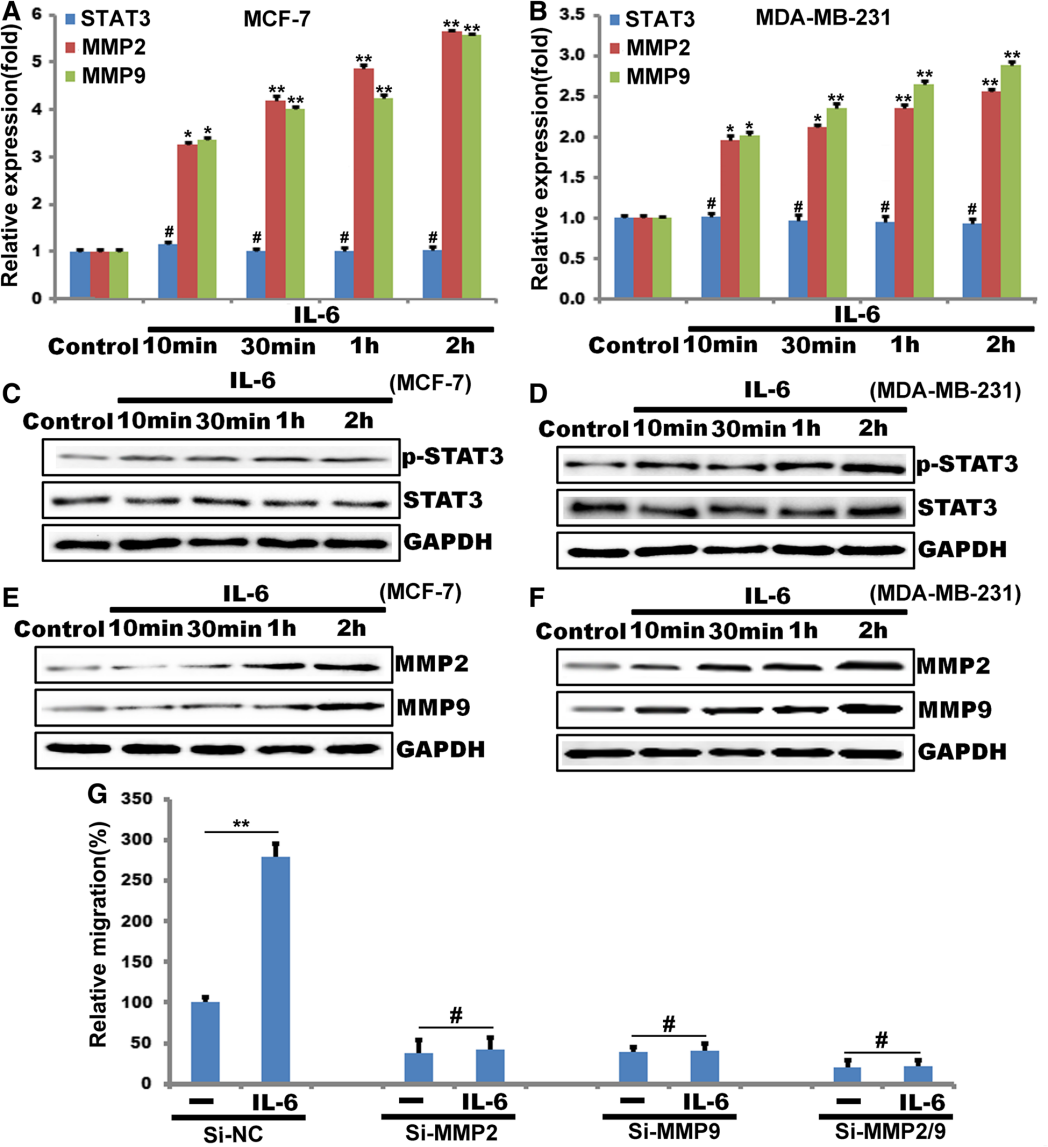
IL-6 induces MMP2 and MMP9 expression in breast cancer cell migration. **a**, **b**, **c** and **d**, MCF-7 and MDA-MB-231 cells were treated with and without IL-6 for the indicated time periods, and either total cellular RNA was isolated and analyzed for STAT3, MMP2 and MMP9 mRNA levels by qPCR (**a** and **b**) or cell extracts were prepared and analyzed for STAT3 phosphotlyation, MMP2 and MMP9 levels by Western blotting using its specific antibodies (**c**, **d** and **e**, **f**). The blot was reprobed for GAPDH for normalization. G, MDA-MB-231 cells were transfected with scrambled (Si-NC) or MMP2 or MMP9 siRNA (100 nM), after 48 h, then cells were treated with or without IL-6 for 2 h, Cell migration was measured by modified Boyden chamber method. (**, *P*<0.01, ^#^, *P*>0.05) *n* = 3

Toward understanding the mechanisms of breast cancer progression, we have previously reported that STAT3 promotes breast cancer cell migration by regulating Cyr61 and Myl9 expression [11]. Interleukin (IL)-6 drives many of the cancer ‘hallmarks’ through downstream activation of the Janus kinase/signal transducer and activator of transcription 3 (JAK/STAT3) signaling pathway [32], we wanted to find whether STAT3 plays a role in IL-6-induced MMP2 and MMP9 expression. IL-6 induced the expression of MMP2 and MMP9 in a biphasic manner at mRNA and protein levels (Fig. 1a, b, e and f) and the phospholyation STAT3(Fig. 1c and d). Based on these observations, we next examined the role of MMP2 and MMP9 in IL-6-induced breast cancer cell migration. Down-regulation of MMP2 expression by siRNA inhibited IL-6-induced breast cancer cell migration about 38%(Fig. 1g) and Si-MMP9 inhibited IL-6-induced breast cancer cell migration about 39%(Fig. G), while Down-regulation of both MMP2 and MMP9 expression by siRNA inhibited IL-6-induced breast cancer cell migration about 20%(Fig. G).

### STAT3 mediates IL-6-induced MMP2 and MMP9 expression and breast cancer cell migration

**Fig. 2:**
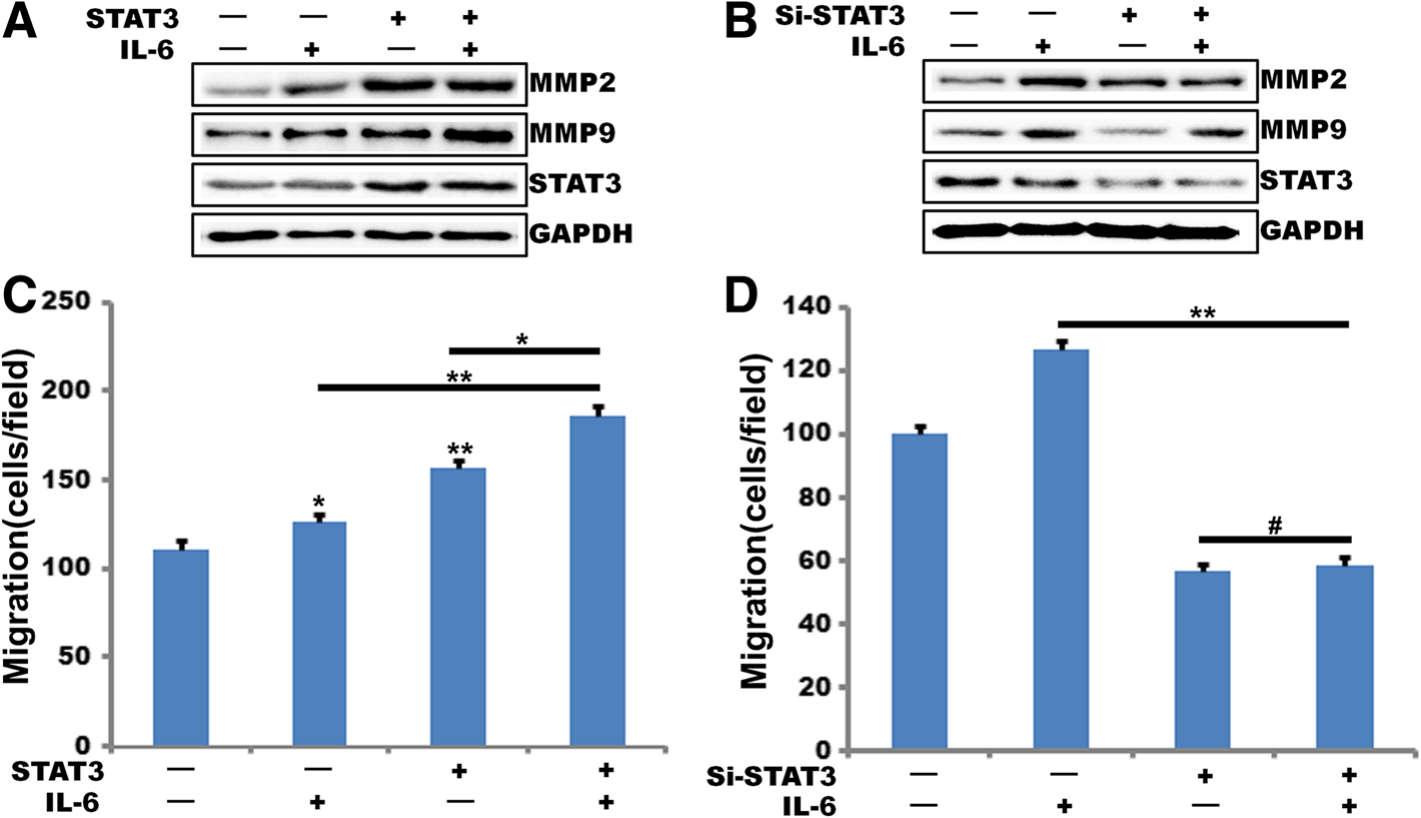
STAT3 mediates IL-6 induced MMP2 and MMP9 expression and breast cancer cell migration. **a** and **b**, MDA-MB-231 cells were either transduced with Ad-GFP or Ad-GFP-STAT3(**a**) or transfected with scrambled (Scr) or STAT3 siRNA (100 nM) (**b**), After 48 h, cells were treated with or without IL-6 for 2 h, and cell extracts were prepared. And analyzed by Western blotting for MMP2 and MMP9 levels using its specific antibodies. The blot was re-probed with anti-GAPDH antibodies for normalization. **c** and **d,** all the conditions were the same as in **a** or **b** except that quiescent MDA-MB-231 cells were subjected to IL-6-induced migration assay (C and D). (**, *P*<0.01, ^#^, *P*>0.05) n = 3

To test the role of STAT3 in IL-6 induced MMP2 and MMP9 expression, we overexpress STAT3 transfecting the cells with plasmid pCDNA3.1-STAT3, over-expression STAT3 significantly induced IL-6-induced MMP2 and MMP9 expression (Fig. 2a). To further verify the role of STAT3 in IL-6-induced MMP2 and MMP9 expression, we transfect the cells with specific STAT3 siRNA, results showed that depletion of STAT3 levels by siRNA suppressed IL-6 induced MMP2 and MMP9 expression (Fig. 2b). These results indicate that STAT3 mediates IL-6-induced MMP2 and MMP9 expression. To understand the role of STAT3 in IL-6-induced breast cancer cell migration, MDA-MB-231 cells that were transduced with either Ad-GFP or Ad-GFP-STAT3, the cells were then treated with and without IL-6, cell migration was measured by a modified Boyden chamber method. Results showed that overexpression of STAT3 enhanced MDA-MB-231 cell migration about 50% (Fig. 2c). In addition, depletion of STAT3 levels by its siRNA also blocked IL-6 induced MDA-MB-231 cell migration about 60% (Fig. 2d). These observations indicate that IL-6-induced MDA-MB-231 cell migration and proliferation require STAT3-mediated MMP2 and MMP9 expression.

### ERα-36 and STAT3 mediate IL-6-induced MMP2 and MMP9 promoter activity

**Fig. 3:**
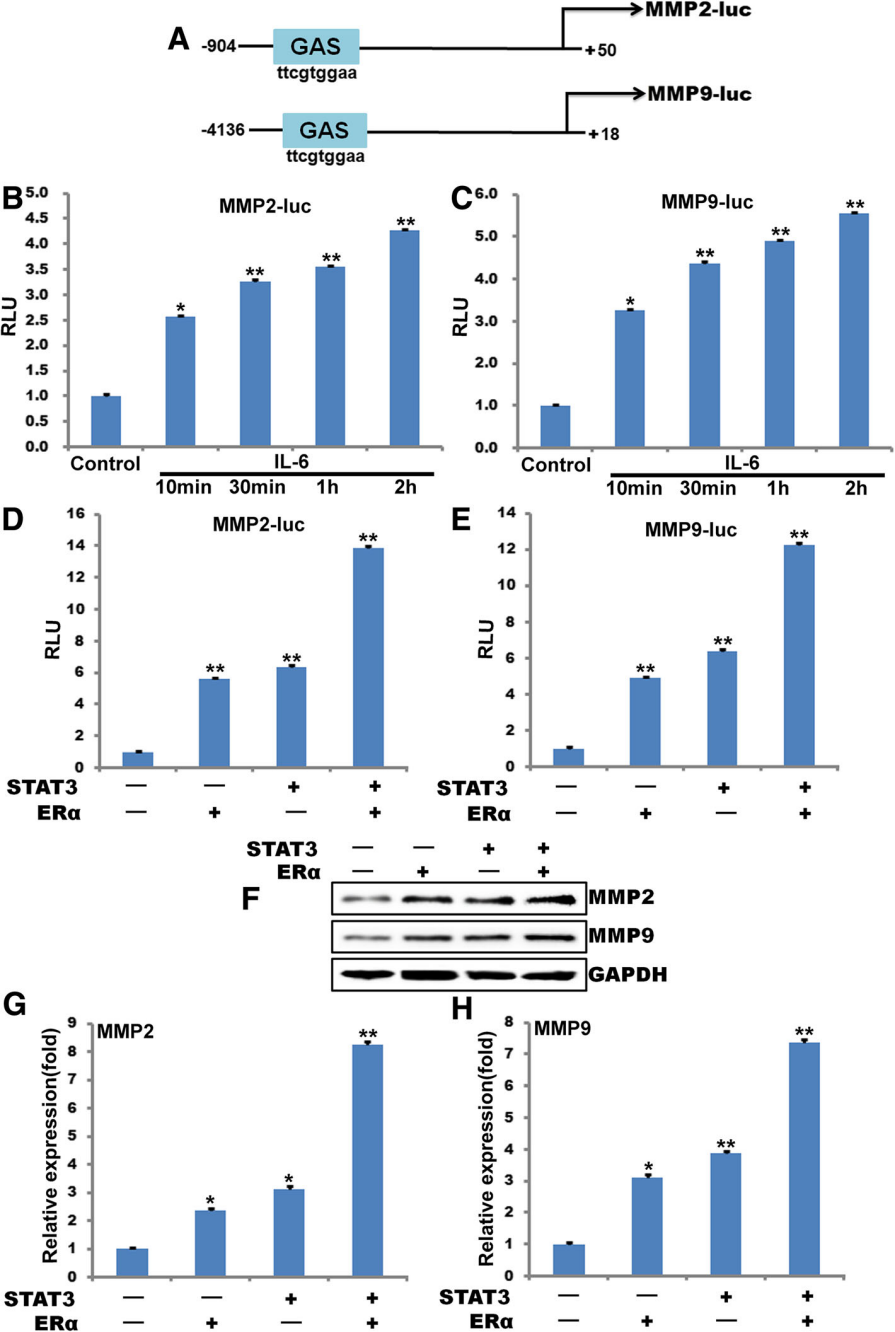
ERα and STAT3 mediates IL-6 induced MMP2 and MMP9 promoter activity **a,** Schematic of the − 904 MMP2 promoter and − 4136 MMP9 promoter, containing GAS element was used for luciferase reporter assay. **b** and **c**, After MDA-MB-231 cells were treated with IL-6 for the indicated time periods. Then the luciferase reporter assays was used to test the transcriptional activity of MMP2 (**b**) and MMP9 (**c**). **d** and **e**, After MDA-MB-231 cells were transfected with ERα-36 or STAT3 for 24 h. Then the luciferase reporter assays was used to test the tanscriptional activity of MMP2 (**d**) and MMP9 (**e**). **f,** 24 h post-transfected with ERα-36 or STAT3, total proteins were extracted and the protein level of MMP2 and MMP9 were detected by Western blotting analysis. GAPDH was used as a loading control. **g** and **h,** MDA-MB-231 cells were transfected with ERα-36 or STAT3 for 24 h, total RNA was isolated and the expression of MMP2 and MMP9 were examined by qPCR. GAPDH was used to serve as a loading control. (**, *P*<0.01, ^#^, *P*>0.05) *n* = 3

Studies from our laboratory as well as others have shown that MMP2 and MMP9 expression is regulated at both transcriptional and post-transcriptional levels [14, 15]. To show STAT3 regulates IL-6-induced MMP2 and MMP9 expression, we first cloned 804 bp MMP2 and 4136 bp MMP9 promoter and analyzed the sequence for transcription factors binding elements using TRANSFAC [33, 34]. We identified one putative STAT-binding element (GAS) in the MMP2 and MMP9 promoter region (Fig. 3a). To find whether IL-6 induces MMP2 and MMP9 promoter activity, MDA-MB-231 cells were transfected with full or truncated MMP2 and MMP9 promoter-luciferase constructs. Cells were either untreated or treated with IL-6 for 1 h. Cell extracts were prepared and analyzed for luciferase activity. Compared with control, IL-6 induced a 2.5-fold or 3-fold increase in luciferase activity with a full-length pGL3-MMP2-luc and pGL3-MMP9-luc construct, and deletion of sequences from in the promoter region resulted in the loss of a response to IL-6, indicating the presence of the IL-6-responsive elements in this region (Fig. 3b and c). To understand the role of STAT3 and ERα-36 in the regulation of MMP2 and MMP9 promoter activity, breast cancer cells were co-transfected with plasmids expressing STAT3 and ERα-36 together with pGL3-MMP2-luc and pGL3-MMP9-luc constructs. Luciferase activity was measured. Our results showed that STAT3 increased the luciferase activity by about 5.5-fold in scrambled siRNA-transfected control cells, and this increase was enhanced by either overexpress ERα-36 or STAT3, indicating a role for ERα in STAT3-induced MMP2 and MMP9 promoter activity (Fig. 3d and e). Similarly, ERα-36 enhances STAT3-induced MMP2 and MMP9 mRNA and protein level by real time PCR and Western blotting approaches (Fig. 3f, g and h).

### Direct interaction of STAT3 and ERα-36

**Fig. 4:**
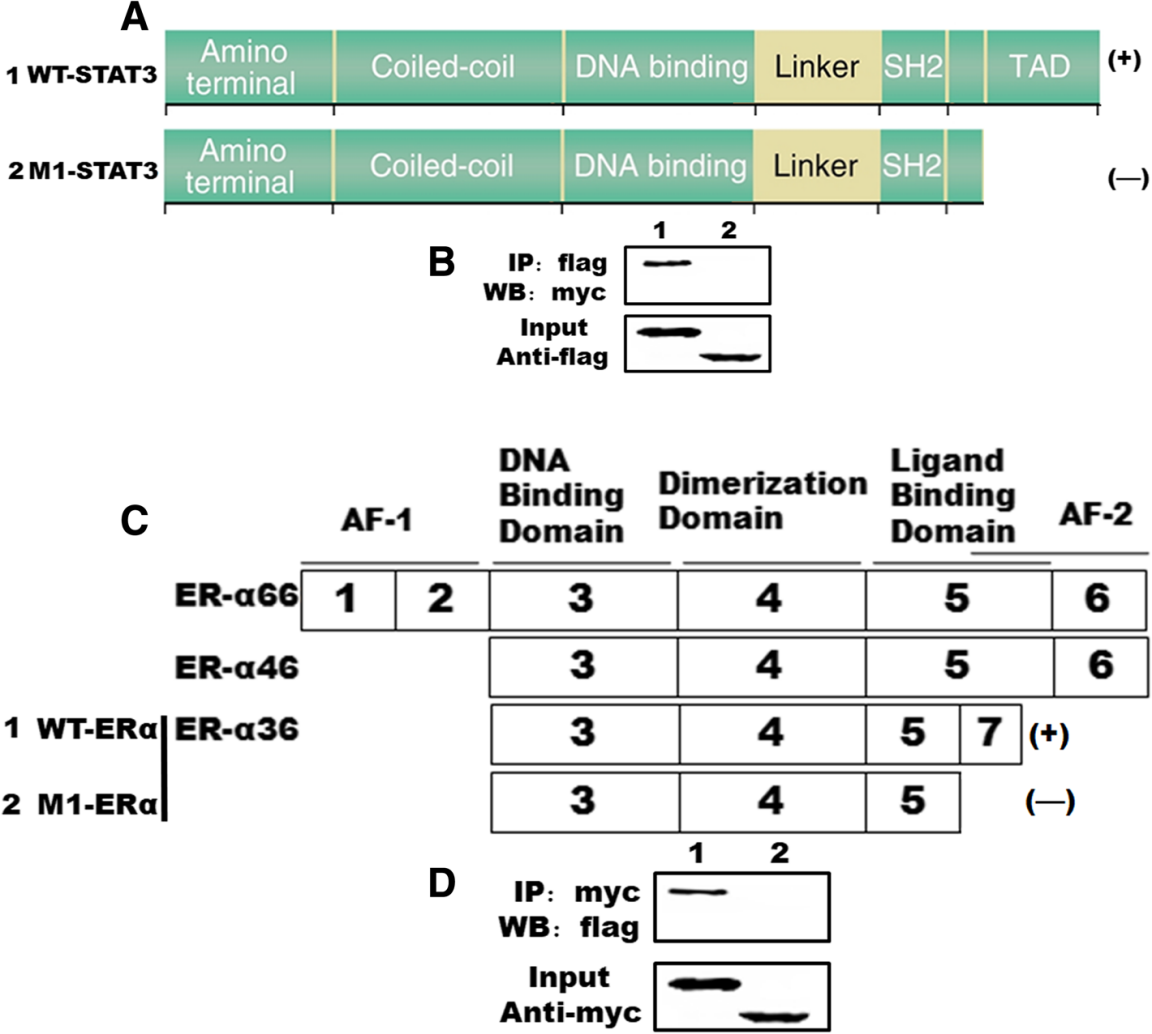
ERα binds to the C-terminus of STAT3. **a**, Schematic diagram of STAT3 and the mutant forms used to map the ERα-36-binding domain. **b**, Coimmunoprecipitation assays. MDA-MB-231 cells were transiently transfected with expression vectors encoding Flag-tagged STAT3 deletion mutant proteins and Myc-tagged ERα-36. Flag-tagged STAT3 deletion mutant proteins were immunoprecipitated (IP) from cell lysates with a monoclonal anti-Flag antibody, and coimmunoprecipitating ERα-36 was detected by immunoblotting (IB) with a monoclonal anti-Myc antibody (top parts). The membrane was re-probed with anti-Flag antibody to reveal the total amount of Flag-tagged STAT3 proteins (bottom parts). **c**, Schematic diagram of ERα-36 and the mutant forms used to map the STAT3-binding domain. **d**, Co-immunoprecipitation assays. MDA-MB-231 cells were transiently transfected with expression vectors encoding Myc-tagged ERα-36 deletion mutant proteins and Flag-tagged STAT3. Myc-tagged ERα-36 deletion mutant proteins were immunoprecipitated (IP) from cell lysates with a monoclonal anti-Flag antibody, and coimmunoprecipitating STAT3 was detected by immunoblotting (IB) with a monoclonal anti-Flag antibody (top). The membrane was re-probed with anti-Myc antibody to reveal the total amount of Myc-tagged ERα-36 proteins (bottom)

We next investigated whether ERα-36 and STAT3 proteins interact by coimmunoprecipitation assays with ERα-36 and STAT3 overexpressed MDA-MB-231 cells. As shown in Fig. 4b and d, ERα-36 specifically interacted with STAT3. The domains of ERα-36 and STAT3 that mediate their interaction were mapped by coimmunoprecipitation assays with a series of ERα-36 and STAT3 deletion mutant proteins (Fig. 4c and d). With a series of STAT3 deletion mutant proteins, we found that mutant proteins lacking sequences C-terminal region of STAT3 lose the ability to interact with ERα-36 (Fig. 4a and b). Similarly, with a series of ERα-36 deletion mutant proteins, we found that mutant proteins lacking sequences C-terminal region of ERα-36 lose the ability to interact with STAT3 (Fig. 4c and d).

### ERα-36 associates with STAT3 and influences STAT3 phospholyation and acetylation

**Fig. 5:**
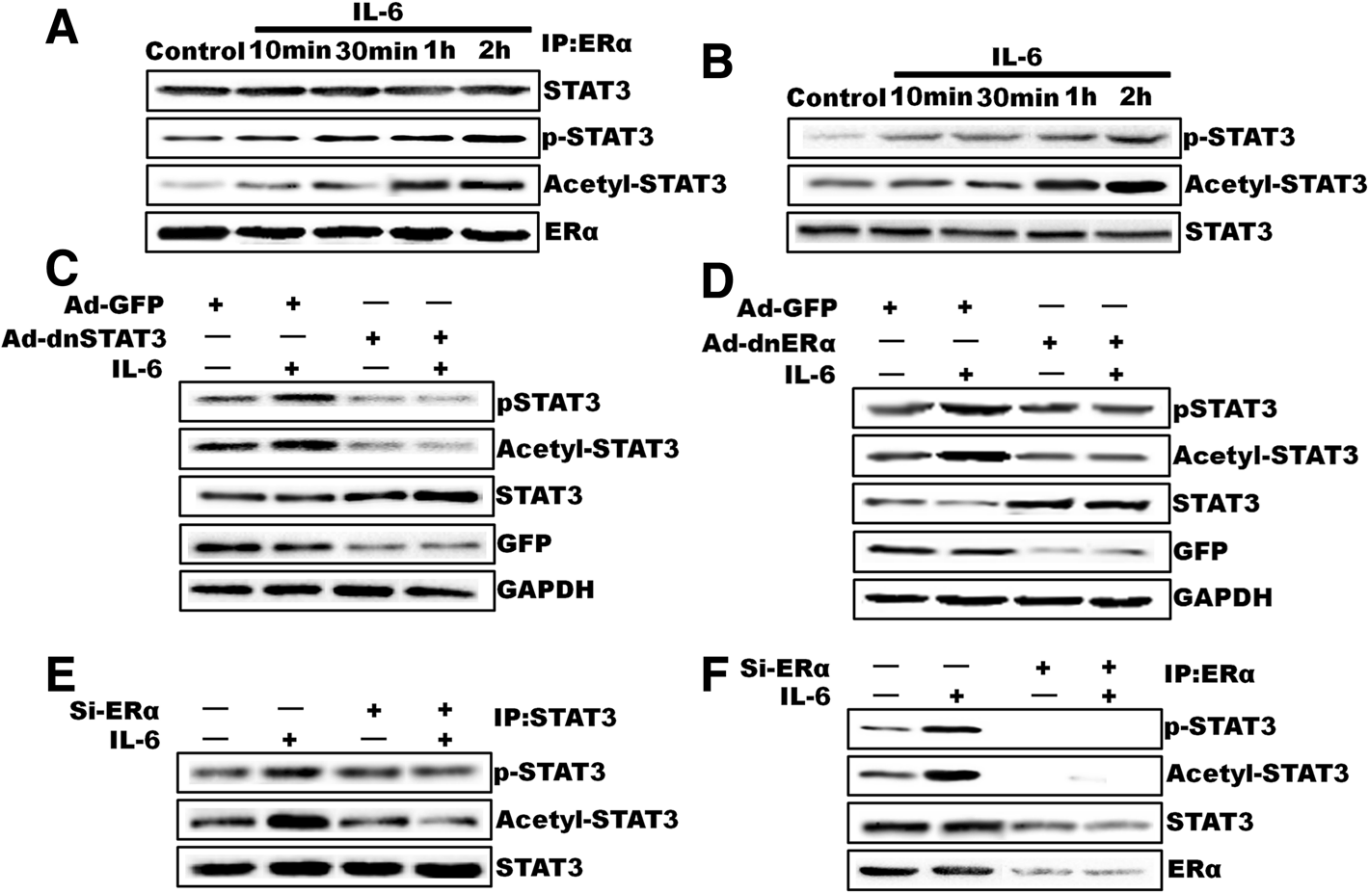
ERα-36 associates with STAT3 and influences STAT3 phosphoryation and acetylation. **a**, quiescent MDA-MB-231 cells were treated with and without IL-6 for the indicated time periods, and cell extracts were prepared. An equal amount of protein from control and each treatment was immunoprecipitated (IP) using anti-ERα-36 antibodies, and the immunocomplexes were analyzed by Western blotting for STAT3 using its specific antibodies. The blot was re-probed sequentially with anti-pSTAT3, anti-acetyl-STAT3, and anti-ERα-36 antibodies. **b**, conditions were same as in A except that an equal amount of protein from control and each treatment was analyzed by Western blotting for STAT3 phosphorylation antibodies. The blot was reprobed sequentially with anti-acetyl-STAT3 and anti-STAT3 antibodies. **c** and **d**, MDA-MB-231 cells that were transduced with Ad-GFP, Ad-dnSTAT3(**c**) Ad-dnERα-36(**d**), after 48 h, cells were treated with and without IL-6 for 1 h, and cell extracts were prepared and analyzed by Western blotting using anti-pSTAT3 and/or anti-acetyl-STAT3 antibodies. The blot was re-probed sequentially with anti-STAT3, anti-GFP, and anti-GAPDH antibodies to show the overexpression of dnSTAT3 or dnERα-36 or for normalization. **e** and **f,** MDA-MB-231 cells that were transfected with scrambled or ERα-36 siRNA (100 nM). 48 h after transfection, cells were treated with or without IL-6 for 1 h, and cell extracts were prepared. An equal amount of protein from control and each treatment was immunoprecipitated with anti-STAT3 (**e**) or anti-ERα-36 (**f**) antibodies, and the immunocomplexes were analyzed by Western blotting using anti-acetyl-STAT3 antibodies. The blot was re-probed sequentially with anti-pSTAT3, anti-STAT3, and/or anti-ERα-36 antibodies

It was well established that STAT3 is activated by phosphorylation at Tyr-705 [35, 36], and its DNA binding as well as transcriptional transactivation activity are enhanced by acetylation at Lys-685 [37]. Hence, we tested whether ERα-36 interaction with STAT3 has any influence on STAT3 phosphorylation or acetylation. Surprisingly, ERα-associated STAT3 was phosphorylated and acetylated with peak effects between 1 and 2 h of IL-6 treatment (Fig. 5a). To find whether there is any connection between STAT3 phosphorylation and its acetylation, first we determined the time course effect of IL-6 on STAT3 phosphorylation (Tyr-705) and acetylation (Lys-685). However, STAT3 is acetylated in a delayed manner peaking between 1 and 2 h of IL-6 treatment (Fig. 5b). Next, we tested the role of ERα-36 on STAT3 phosphorylation and acetylation, we blocked IL-6-induced STAT3 activation by Ad- dominant-negative STAT3(Ad-dnSTAT3) and tested its effect on STAT3 phosphorylation and acetylation. Blockade of ERα-36 activation did inhibit STAT3 phosphorylation and acetylation (Fig. 5c). However, overexpression of Ad- dominant-negative ERα-36 (Ad-dnERα-36) blocked phosphorylation and acetylation at 1 h of IL-6 treatment (Fig. 5d). These observations led to the conclusion that ERα-36 mediates both phosphorylation and acetylation of STAT3 at 1 h upon IL-6 treatment. To further confirm the results, ERα-36 levels were down-regulated by its siRNA, and its effect was tested on IL-6-induced STAT3 phosphorylation and acetylation. Depletion of ERα-36 levels also led to a decrease in IL-6-induced phosphorylation and acetylation of both total and ERα-36 associated-STAT3 (Fig. 5e and f). These results indicate that IL-6-induced STAT3 phosphorylation and acetylation at 1 h of IL-6 treatment require ERα-36 activation.

### STAT3 phospholyation is required for IL-6-induced MMP2 and MMP9 expression and breast cancer cell migration

**Fig. 6:**
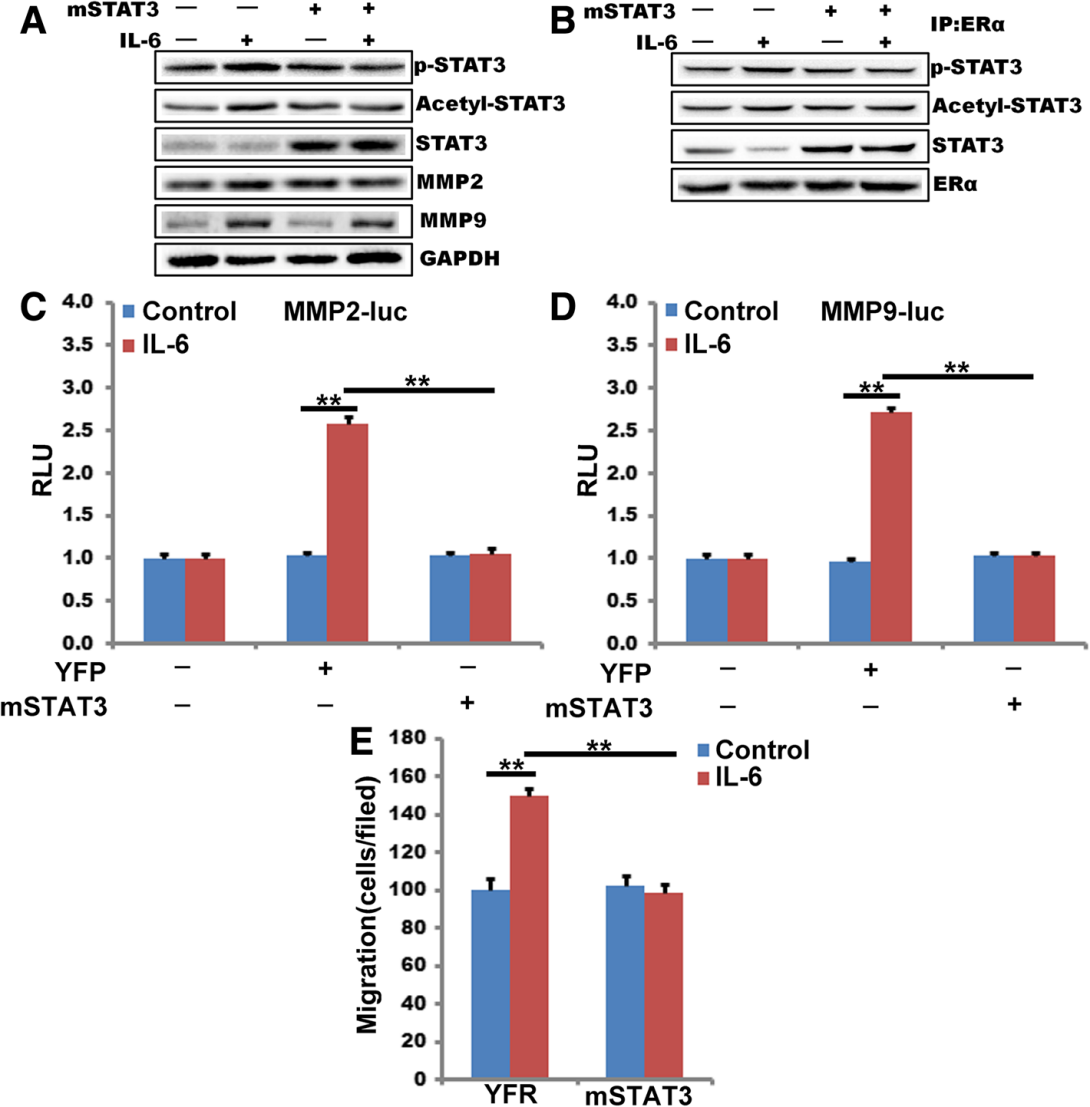
STAT3 phosphotlyation is required for IL-6 induced MMP2 and MMP9 expression and breast cancer cell migration. **a** and **b**, MDA-MB-231 that were transfected with pCDNA3.1-YFP or pCDNA3.1-mSTAT-3, 48 h after transfection, cells were treated with and without IL-6 for 1 h; cell extracts were prepared and analyzed by Western blotting for STAT3 acetylation using anti-acetyl-STAT3 antibodies (**a**) or immunoprecipitated (IP) with anti-ERα-36 antibodies, and the immunocomplexes were analyzed for acetyl-STAT3 as described in A (**b**). IB, immunoblot. Blots were re-probed sequentially with anti-pSTAT3, anti-STAT3, anti-MMP2, anti-MMP9, anti-ERα-36, and/or anti-GAPDH antibodies. **c** and **d,** MDA-MB-231 cells were transfected with pCDNA3.1-YFP or pCDNA3.1-mSTAT3 in combination with either empty vector or pGL3-MMP2-luc (**c**) or pGL3-MMP9-luc (**d**) and analyzed for IL-6-induced luciferase activity. RLU, relative luciferase units. **e,** MDA-MB-231 cells were transfected with pCDNA3.1-YFP or pCDNA3.1-mSTAT3, quiesced and subjected to IL-6-induced MDA-MB-231 cell migration. (**, *P*<0.01, ^#^, *P*>0.05) *n* = 3

To understand the role of STAT3 acetylation on its tyrosine phosphorylation, we used an acetylation null mutant of STAT3, STAT3K685R [38]. Interference of STAT3 acetylation by STAT3K685R blocked both total and ERα-36-associated STAT3 phosphorylation (Fig. 6a and b). This finding indicated that STAT3 acetylation is required for tyrosine phosphorylation. To understand the role of acetylation on IL-6-induced MMP2 and MMP9 expression, MDA-MB-231 cells were cotransfected with STAT3K685R (pCDNA3-mSTAT-3) and pGL3-MMP2-luc and pGL3-MMP9-luc construct, Cell were then treated with or without IL-6 for 1 h. Cell were then analyzed for MMP2 and MMP9 promoter activity. STAT3K685R mutant reduced IL-6-induced MMP2 and MMP9 promoter-luciferase activity about 2.5-fold or 2.7-fold, respectively (Fig. 6c and d). overexpress STAT3K685R also inhibits IL-6-induced breast cancer cell migration about 40% (Fig. 6 E).

### ERα-36 recruits p300 and mediates IL-6-induced STAT3 acetylation

**Fig. 7:**
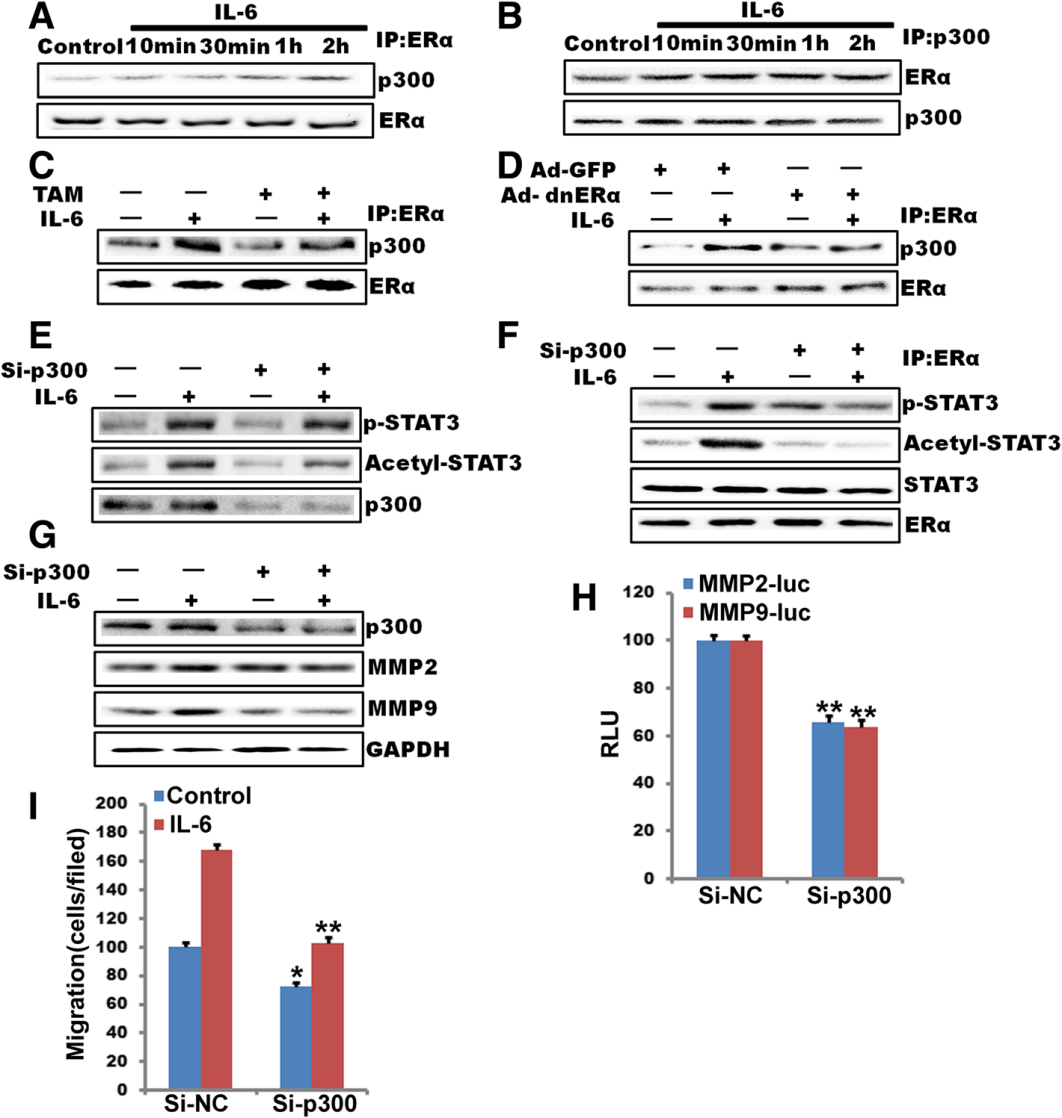
ERα-36 recruits p300 and mediates IL-6 induced STAT3 acetylation. **a** and **b,** quiescent MDA-MB-231 cells were treated with and without IL-6 for the indicated time periods, and cell extracts were prepared. Cell extracts containing an equal amount of protein from control and each treatment were immunoprecipitated (IP) with anti-ERα-36 or anti-p300 antibodies, and the immunocomplexes were analyzed by Western blotting using anti-p300 and/or anti-ERα-36 antibodies. IB, immunoblot. **c**, quiescent MDA-MB-231 cells were treated with and without IL-6 in the presence and absence of TAM (10 μM) for 1 h, and cell extracts were prepared. Cell extracts containing an equal amount of protein from control and each treatment were immunoprecipitated with anti-ERα-36 antibodies, and the immunocomplexes were analyzed by Western blotting using anti-p300 antibodies. **d**, all the conditions were the same as in B except that MDA-MB-231 cells were transduced with Ad-GFP or Ad-dnERα-36 and quiesced before being treated with IL-6. Using with anti-ERα-36 antibodies to show equal amounts of immunoprecipitated target protein. **e**, **f** and **g**, MDA-MB-231 cells that were transfected with scrambled (Scr) or p300 siRNA (100 nM) and quiesced were treated with and without IL-6 for 1 h, and cell extracts were prepared. Cell extracts containing an equal amount of protein from control and each treatment were analyzed by Western blotting using anti-pSTAT-3 and anti-acetyl-STAT3 antibodies (**e**) using anti-MMP2 and anti-MMP9 (**g**) antibodies or subjected to immunoprecipitation using anti-ERα antibodies followed by immunoblotting using anti-acetyl-STAT3 antibodies (**f**). The blots were reprobed sequentially with anti-pSTAT3, anti-STAT3, and anti-ERα-36 antibodies. **h,** MDA-MB-231 cells that were transfected with scrambled or p300 siRNA (100 nM) in combination with either empty vector or pGL3-MMP2-luc and pGL3-MMP9-luc, and cell extracts were prepared and analyzed for luciferase activity. RLU, relative luciferase units. **i,** MDA-MB-231 cells that were transfected with scrambled (Scr) or p300 siRNA (100 nM) and quiesced were treated with and without IL-6 for 1 h induced MDA-MB-231 cell migration. Bar graphs represent means ±S.D. values of three independent experiments. (**, *P*<0.01, ^#^, *P*>0.05) *n* = 3

Previous studies have demonstrated that STAT3 acetylation is mediated by p300 [39]. We propose a hypothesis that ERα-36 may recruit p300 and thereby facilitate ERα-36-induced STAT3 acetylation. To test this hypothesis, we studied the association of ERα-36 and p300 in control versus IL-6-treated MDA-MB-231 by coimmunoprecipitation. As expected, p300 was associated with ERα-36 in response to IL-6 (Fig. 7a). Maximum increase in ERα-36 and p300 association was detected between 1 and 2 h of IL-6 treatment, and this was correlated with the time course of STAT3 acetylation. In addition, blockade of ERα-36 activation by TAM (10 M), or Ad-dnERα-36 resulted in the loss of ERα-36 association with p300 (Fig. 7b, c and d). Moreover, knockdown of p300 levels by its siRNA also reduced IL-6-induced total and ERα-36-associated STAT3 acetylation and its late phase phosphorylation (Fig. 7e, f and g). In addition, down-regulation of p300 levels attenuated IL-6- induced MMP2 and MMP9 promoter activity and its expression about 38% or 40%, respectively (Fig. 7h). These observations strongly support a role of ERα-36-recruited p300 in IL-6-induced STAT3 acetylation and phosphorylation, leading to MMP2 and MMP9 expression. To understand the functional of p300, we also tested its role in IL-6-induced breast cancer cell migration and proliferation. Down-regulation of p300 levels by its siRNA attenuated IL-6-induced breast cancer cell migration about 60% (Fig. 7i).

### ERα-36 and STAT3 bind to MMP2 and MMP9 promoter and mediate MMP2 and MMP9 expression in response to IL-6

**Fig. 8:**
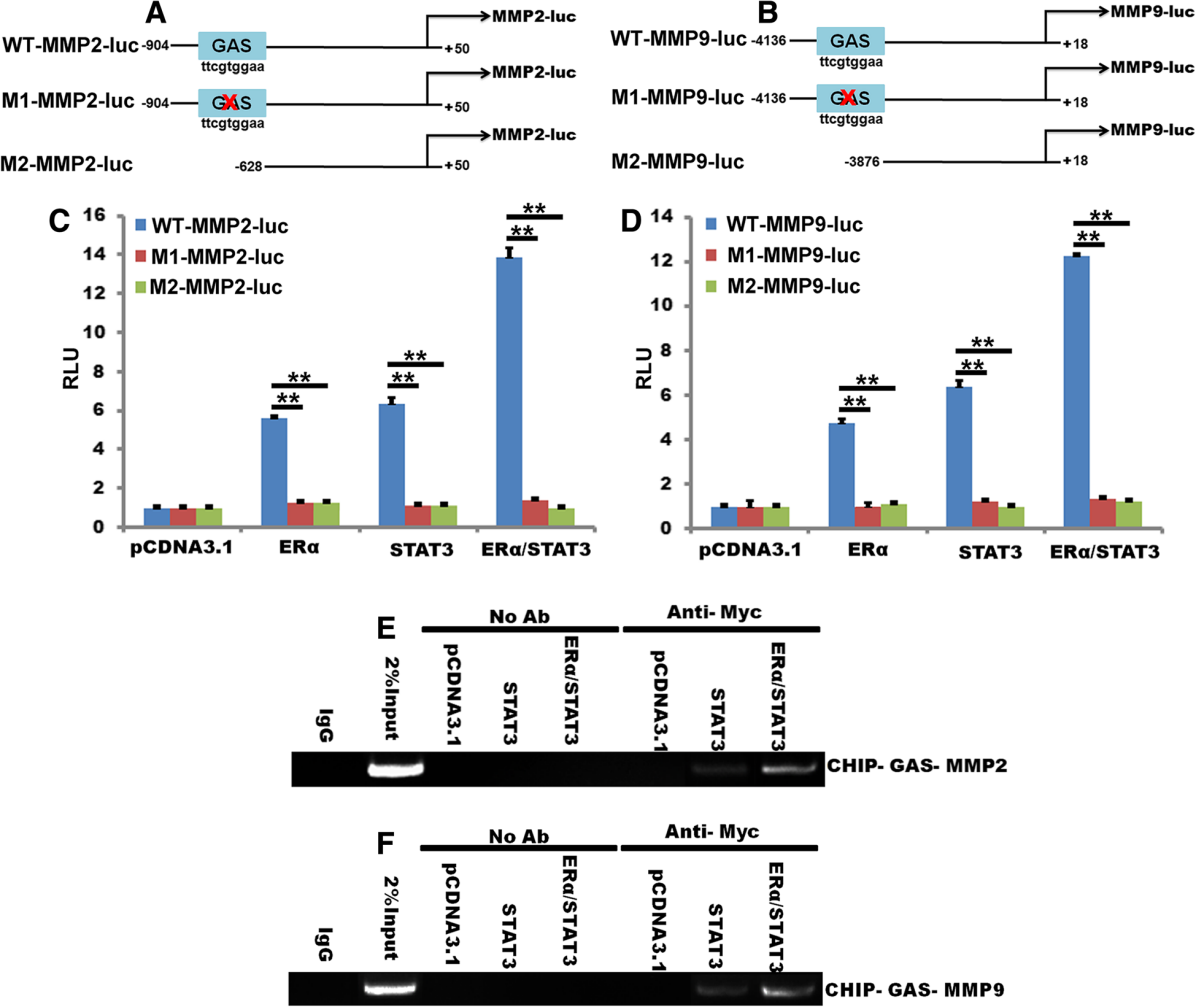
ERα-36 and STAT3 bind to MMP2 and MMP9 promoter and mediate MMP2 and MMP9 expression in response to IL-6. **a,** Schematic of the − 904 MMP2 promoter, containing GAS element was linked to a luciferase reporter. Mutation or truncations that remove the GAS element. -904 MMP2 promoter, a truncated promoter − 828 or the − 904 MMP2 promoter with mutations in GAS. **b,** Schematic of the − 4138 MMP9 promoter, containing GAS element was linked to a luciferase reporter. Mutation or truncations that remove the GAS box element. -4138 MMP9 promoter, a truncated promoter − 3876 or the − 4138 MMP9 promoter with mutations in GAS. **c,** MDA-MB-231 cells were transfected with the wild-type − 904 MMP2 promoter, or a truncated promoter − 828 or the − 904 MMP2 promoter with mutations in GAS, and transfected with ERα-36 or STAT3 48 h. Then the luciferase reporter assays was used to test the transcriptional of MMP2. **d,** MDA-MB-231 cells were transfected with the wild-type − 4138 MMP9 promoter, or a truncated promoter − 3876 or the-4138 MMP9 promoter with mutations in GAS, and transfected with ERα-36 or STAT3 48 h. Then the luciferase reporter assays was used to test the transactivity of MMP9. **e** and **f,** MDA-MB-231 cells were transiently transfected with a STAT3 ERα-36/STAT3 or a control vector (pCDNA3.1) 48 h, and ChIP assays were performed as described in Materials and Methods with primers for sequences associated with the genes for MMP2 and MMP9. Sheared DNA/protein complexes were immunoprecipitated by using an anti-Myc-STAT3 Ab. Then, PCR was carried out to detect the endogenous GAS regions in immunoprecipitated chromatin fragments. The amount of DNA in each sample (2%input) is shown at the second land. Immunoprecipitations were performed without primary antibody (No Ab) as a control and IgG as a negative control

To identify the critical promoter elements responsible for the effect of STAT3, ERα and their synergy. The proximal portion of the MMP2 and MMP9 promoter contains several regulatory elements, including one GAS box. To characterize the importance of these elements, we generated a set of luciferase reporter constructs with various mutations of the MMP2 and MMP9 promoter (Fig. 8a and b). Promoter luciferase assay showed both STAT3 and ERα-36 induced a modest increase in the promoter activity, whereas the combined treatment acted synergistically (Fig. 8c and d). Interestingly, inactivating mutations of this element alone both inhibited the effect of the individual treatments and affected their synergy. Importantly, Mutation of the GAS had an almost complete inhibitory effect (Fig. 8c and d). Together, these findings indicate that the GAS is necessary and sufficient not only for ER-mediated activation of the promoter but also for the STAT3-triggered response and synergy between these signals.

ChIP assays were used and further confirmed the impact of STAT3 and ERα-36 on the MMP2 and MMP9 gene promoters. Our results show that STAT3 binds the GAS of the MMP2 and MMP9 promoter, ERα-36 enhances this effect (Fig. 8e and f) and STAT3 bind the GAS of the MMP2 and MMP9 promoter. These results confirmed that GAS is necessary and sufficient for STAT3 and ERα-36 mediated MMP2 and MMP9 promoter activity in MDA-MB-231 cells.

### MMP2 and MMP9 cellular location in response to IL-6

**Fig. 9:**
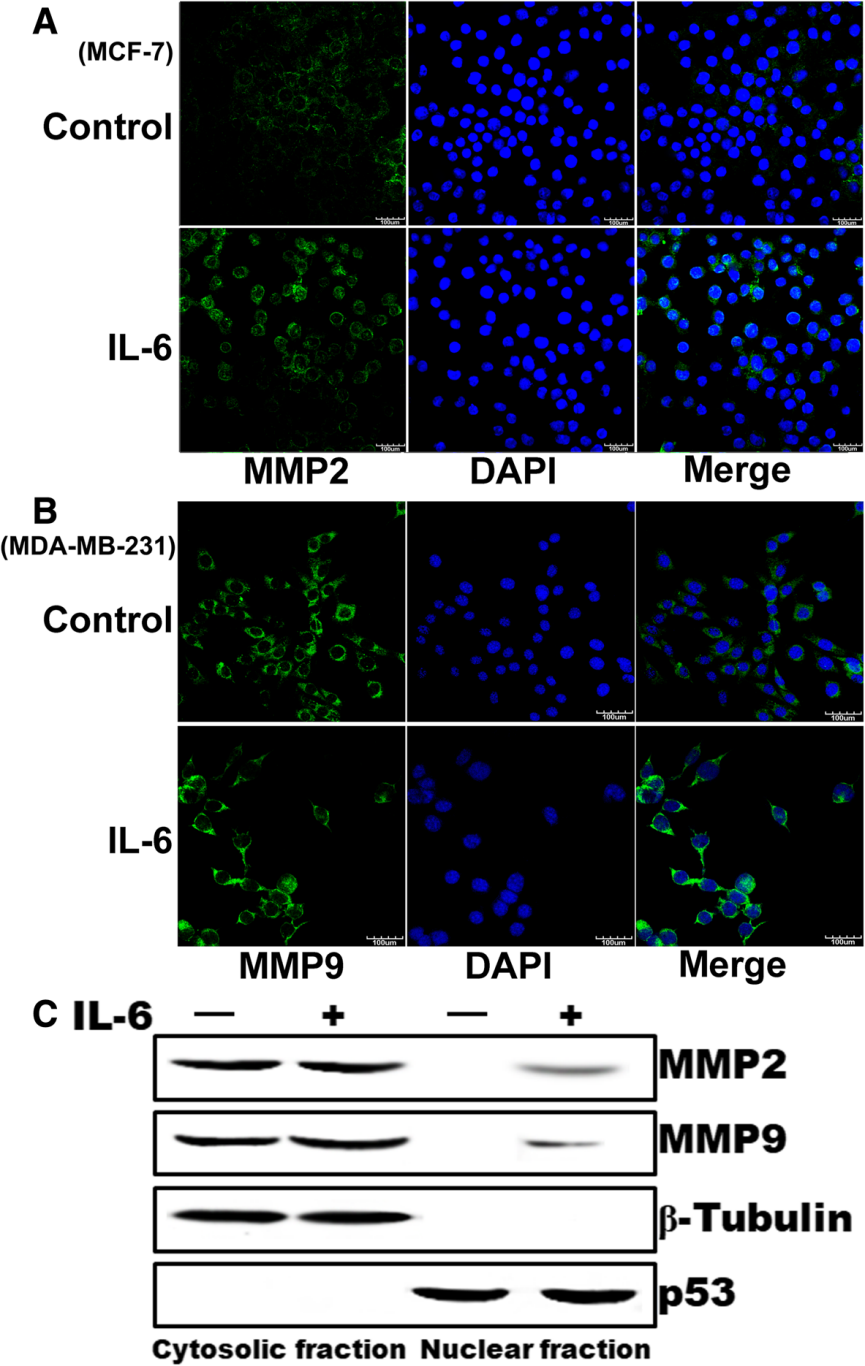
Translocation of MMP2 and MMP9 the cytoplasm to the nucleus in response to IL-6. **a** and **b,** quiescent MDA-MB-231 cells were treated with and without IL-6 for the indicated time periods, fixed, permeabilized, and immunostained for MMP2 and MMP9 using its specific antibodies followed by probing with Alexa Fluor 488-conjugated secondary antibodies. **c**, quiescent MDA-MB-231 cells were treated with and without IL-6 for 1 h and the cytoplasmic and nuclear extracts were prepared. An equal amount of protein from the cytoplasmic and nuclear extracts of control and the IL-6-treated MDA-MB-231 cells was analyzed by Western blotting for MMP2 and MMP9 levels using its specific antibodies. The blot was reprobed sequentially with anti-β-tubulin and anti-p53 antibodies to show the relative purity of the cytoplasmic and nuclear extracts

To test the cellular location of MMP2 and MMP9, we conducted immunofluorescence staining studies to observe the localization of MMP2 and MMP9 during the IL-6-induced expression. As seen in Fig. 9a and b, MMP2 and MMP9 were detected in the cytoplasm without IL-6 treatment and translocated to the nucleus treated with IL-6. To further confirm this result, we prepared cytoplasmic and nuclear extracts from IL-6-treated MDA-MB-231 cells and analyzed for MMP2 and MMP9 levels in both the fractions. MMP2 and MMP9 were present in the cytoplasmic fraction at without IL-6 treatment. After beening treated with IL-6, MMP2 and MMP9 were detected in both the fractions (Fig. 9c). These results provide further clues supporting our hypothesis that IL-6 regulates the MMP2 and MMP9 expression, during which it is mostly located in the nuclear and mediate cell migration.

### ERα-36 and STAT3 signaling cross-talking human breast cancer cell migration

**Fig. 10:**
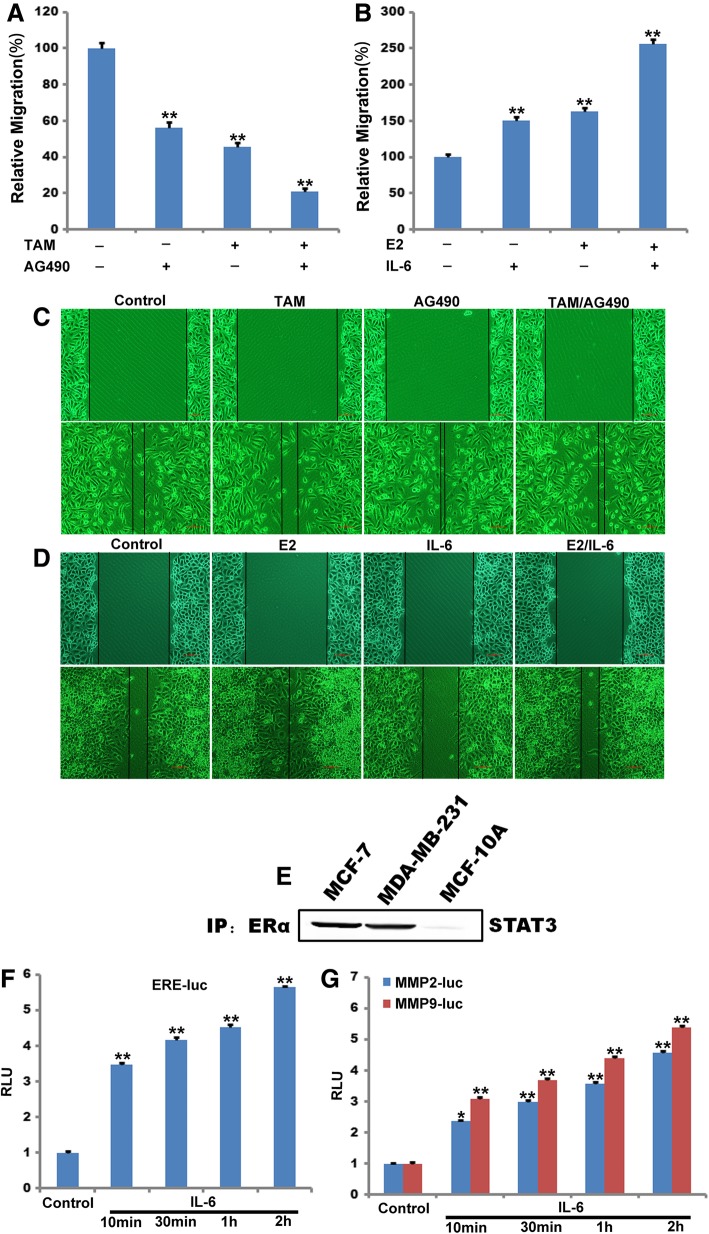
ERα and JAK2-STAT3 signaling cross-talking human breast cancer cell. **a** and **b**, Transwell assay was used to detect the migration-stimulating effects of TAM and AG490 or E2 and IL-6 in MDA-MB-231 cells. The number of cells migrated to the lower side of the transwell chambers was counted and photographed in five fields (the upper, the lower, the left, the right, and the middle) of three independent experiments, and the fold migration was calculated. (The negative control (DMSO) of Fig. 10a and the negative control (DMSO/PBS) of Fig. 10b) (*P < 0.05, **P < 0.01.n = 3). **c** and **d**, The effect of overexpressed TAM and AG490 or E2 and IL-6 on MDA-MB-231 migration of was detected by Wound-healing assay. **E,** MDA-MB-231, MCF-7 and MCF-10A cell lysates were immunoprecipitated with an anti-ERα antibody and probed with an anti-STAT3 antibody. **f** and **g,** Effects of IL-6 on ERE transcription activity (**f**) and effects of IL-6 on MMP2 and MMP9 transcription activity (**g**) were investigated in MDA-MB-231 cells. The luciferase activity was expressed as a percentage relative to the control was used. All luciferase assays were performed in duplicates. (*P < 0.05, **P < 0.01.n = 3)

Since both E2 and IL-6 play crucial roles in mammary gland development, differentiation and tumorigenesis, it would be revealing to see whether the ER-STAT3 cross-talk actually takes place in cells of mammary gland origin. Wound-healing assay data show that the wound treated with ER signal inhibitor TAM, AG490 decreased the rate of wound closure, compare to the control group. More importantly, E2 and IL-6 can synergistically promote the healing of breast cancer cells scratch and migration. (Fig. 10a, b, c and d). Then, three ER expressing human breast cancer cell lines, MDA-MB-231, MCF-7 and MCF10A were used to study such events. Direct association of ERα-36 and STAT3 was identified in lysates from both cell lines by co-immunoprecipitation studies (Fig. 10e). For the functional assays, both cells were transfected with the ERE-LUC reporter plasmid and treated with E2 alone, or E2 plus IL-6, to investigate whether addition of IL-6 will influence the ER signaling. Data from this assay indicated that addition of IL-6 induced ER signaling in both cell lines (Fig. 10f). To assess the effect of ER activation on JAK-STAT3 signaling, cells were transfected with the MMP2 and MMP9-luc reporter plasmid and treated with IL-6 alone, or IL-6 plus E2. The results showed that addition of E2 enhanced IL-6 signaling in MDA-MB-231 cells (Fig. 10g).

## Discussion

The pathogenic roles of MMPs in human diseases such as cancer have attracted great attention. The most straightforward explanation for their role in cancer is that MMPs, through extracellular matrix degradation, pave the way for tumor cell invasion and metastasis [16]. Increasing evidence suggests that high levels of serum IL-6 are associated with poor prognosis, advanced disease, and metastases in breast cancer patients [40, 41]. Our results in this study also showed that IL-6 induced MMP2 and MMP9 expression in breast cancer cell and mediated cell migration.

Previous reports from us and others indicated that STAT3 could promote the proliferation and metastasis of breast cancer cells by EMT pathway [11, 13, 30]. In this study, we found that STAT3 could also mediate IL-6-induced MMP2 and MMP9 expression and breast cancer cell migration. Estrogen receptor alpha could mediated proliferation of human breast cancer via a p21/PCNA/E2F1-dependent pathway [42, 43]. ERα-36 is a novel variant of ER-α that have a molecular weight of 36-kDa. This ERα variant differs from the original 66 kDa ERα (ERα-66), lacking both transcriptional activation domains (AF-1 and AF-2) but retaining the DNA-binding domain and partial dimerization and ligand-binding domains [44]. Our data showed that ERα-36 and STAT3 also mediated IL-6-induced MMP2 and MMP9 promoter activity. We also proved that ERα-36 was also associated with STAT3 and influenced phospholyation and acetylation of STAT3, which was required for IL-6-induced MMP2 and MMP9 expression and breast cancer cell migration. And Cross-talk between ERα-36 and STAT3 was demonstrated to mediate through a direct physical association between the two proteins. By coimmunoprecipitation assays with various constructs of ERα-36 and STAT3, it was shown that the C-termini of these two proteins were mainly responsible for this interaction.

STAT3 is also known to be acetylated (Ac) on multiple lysine (K) residues by the CBP/p300 histone acetyltransferase in response to cytokines and growth factor signaling. In contrast, recently study showed that insulin stimulated K87 acetylation promote STAT3 mitochondrial translocation and functions [39, 45]. Despite the classical activation of p-STAT3 by JAK2, STAT3 acetylation by p300/CBP histone acetyltransferase (HAT) is also essential for STAT3 to form stable dimers and to activate STAT3 target gene transcription, acetylated STAT3 forms stable dimer to translocate into nucleus and binds strongly to the DNA to enhance the transcription of STAT3 target genes [38, 46]. In this study, we proved that ERα-36 recruited p300 and mediated IL-6-induced STAT3 acetylation. In additional, ERα-36 and STAT3 bind to MMP2 and MMP9 promoter and mediate MMP2 and MMP9 expression in response to IL-6. MMP2 and MMP9 translocated from the cytoplasm to the nuclear in response to IL-6. ERα-36 and STAT3cross-talk during human breast cancer cell migration, this provides a novel pathway in breast cancer progression.

## Conclusions

Our studies demonstrate for the first time that the function of MMP2 and MMP9 in breast cancer cell migration, which is mediated by interactions between ERα-36 and STAT3.

## Data Availability

The data generated during this study are included in this article and its supplementary information files are available from the corresponding author on reasonable request.

## Abbreviations

AF-1: Transactivation function
AF-2: Ligand-dependent transactivation
BRMS1: Breast cancer metastasis suppressor 1
DAPI: 4′,6-diamidino-2-phenylindole
DBD: DNA-binding domain
DMEM: Dulbecco’s modified Eagle’s medium
EB: Ethidium bromide
EMT: Epithelial to mesenchymal transition
ER: Estrogen receptor
ERE: Estrogen response elements
HAT: Histone acetyltransferase
LBD: Ligand binding domain
OPN: Osteopontin
uPA: Plasminogen activator
